# Detection and quantification of epithelial progenitor cell populations in human healthy and IPF lungs

**DOI:** 10.1186/s12931-016-0404-x

**Published:** 2016-07-16

**Authors:** N. F. Smirnova, A. C. Schamberger, S. Nayakanti, R. Hatz, J. Behr, O. Eickelberg

**Affiliations:** Comprehensive Pneumology Center, Institute of Lung Biology and Disease, University Hospital, Ludwig-Maximilians-University and Helmholtz Zentrum München, Max-Lebsche-Platz 31, Munich, 81377 Germany; Thoraxchirurgisches Zentrum, Klinik für Allgemeine, Viszeral, Transplantations, Gefäß- und Thoraxchirurgie, Klinikum Großhadern, Ludwig-Maximilians-Universität, Munich, Germany; Asklepios Fachkliniken München-Gauting, Munich, Germany; Medizinische Klinik und Poliklinik V, Klinikum der Ludwig-Maximilians-Universität, Munich, Germany; German Center of Lung Research (DZL), Hannover, Germany; Comprehensive Pneumology Center (CPC), Ludwig-Maximilians-University and Helmholtz Zentrum München, Max-Lebsche-Platz 31, Munich, D-81377 Germany

**Keywords:** Epithelium, Progenitor cells, Basal cells, Subpopulations, Human explants

## Abstract

**Background:**

In the human lung, epithelial progenitor cells in the airways give rise to the differentiated pseudostratified airway epithelium. In mice, emerging evidence confers a progenitor function to cytokeratin 5 (KRT5^+^) or cytokeratin 14 (KRT14^+^)-positive basal cells of the airway epithelium. Little is known, however, about the distribution of progenitor subpopulations in the human lung, particularly about aberrant epithelial differentiation in lung disease, such as idiopathic pulmonary fibrosis (IPF).

**Methods:**

Here, we used multi-color immunofluorescence analysis to detect and quantify the distribution of airway epithelial progenitor subpopulations in human lungs obtained from healthy donors or IPF patients.

**Results:**

In lungs from both, healthy donors and IPF patients, we detected KRT5^+^KRT14^-^, KRT5^-^KRT14^+^ and KRT5^+^KRT14^+^ populations in the proximal airways. KRT14^+^ cells, however, were absent in the distal airways of healthy lungs. In IPF, we detected a dramatic increase in the amount of KRT5^+^ cells and the emergence of a frequent KRT5^+^KRT14^+^ epithelial population, in particular in distal airways and alveolar regions. While the KRT14^-^ progenitor population exhibited signs of proper epithelial differentiation, as evidenced by co-staining with pro-SPC, aquaporin 5, CC10, or MUC5B, the KRT14^+^ cell population did not co-stain with bronchial/alveolar differentiation markers in IPF.

**Conclusions:**

We provide, for the first time, a quantitative profile of the distribution of epithelial progenitor populations in human lungs. We show compelling evidence for dysregulation and aberrant differentiation of these populations in IPF.

## Background

Lung function, barrier integrity, and epithelial homeostasis are maintained, in large parts, by its inner lining, which is constituted by lung epithelial cells. Different types of lung epithelial cells have been identified and characterized in the past, including ciliated, goblet, or basal cells in proximal airways, and ciliated, club (Clara), neuroendocrine, or basal cells in distal airways. The pseudostratified airway epithelial layer continues into a flat epithelial layer composed of alveolar type 1 (AT1) or type 2 (AT2) cells in the alveolar regions. Lung injury induced by injurious agents (e.g. cigarette smoke, particulate matter, viruses) primarily affects the integrity of this epithelial compartment, either by inducing barrier dysfunction or epithelial progenitor cell perturbation [1–4].

Recently, lung epithelial progenitor cells have attracted significant interest, as they serve as stem cells for differentiated lung epithelial cell types, such as secretory, ciliated, or AT1 cells in mice [3]. Basal cells are relatively undifferentiated epithelial cells, which are located attached to the basal lamina of the stratified and pseudostratified airway epithelium [5]. Basal cells participate in anchoring of the airway epithelium to the basement membrane and thus separate the underlying stroma from the epithelial tubes. Airway basal cells also maintain the homeostasis of the normal epithelial layer by giving rise to the differentiated airway epithelial cells during postnatal growth and in the adult under steady state, as previously shown by us and others using a variety of in vivo and in vitro assays [6–8].

Airway basal cell dysfunction contributes to a number of pathologies, as disruption in the balance between basal cell proliferation and differentiation leads to epithelial hyperplasia/hypoplasia [5] or abnormal differentiation patterns [4, 9], e.g. in lung cancer. Hyperplastic basal cell lesions are also observed in chronic obstructive pulmonary disease (COPD) [5, 10] and cystic fibrosis (CF) [11], whereas the epithelial injury and loss in bronchiolitis obliterans syndrome (BOS) may indicate stem cell exhaustion or a loss in epithelial progenitor populations [5].

In mice, recent studies using a number of reporter and fate-mapped mouse strains have demonstrated that lung basal cells contribute to regeneration of the lung in response to injury, based on their capacity to proliferate and differentiate into mature cell types [12, 13]. Evidence of the regenerative capacity of basal cells in human results from the observation that basal cells isolated from different regions of the human lung proliferate and differentiate to mature ciliated, goblet, or club (Clara) cells in air-liquid interface (ALI) cultures or bronchosphere assays *ex vivo* [14]. In vivo, injury/repair models have demonstrated that disruption of the basal cell layer is associated with an uncontrolled proliferation of the underlying stroma, resulting in an accumulation of fibroblasts and immune cells that subsequently obliterate the airways [15].

Emerging evidence shows that basal cells are composed of multiple heterogeneous subpopulations, under physiological as well as pathological conditions. As an example, mouse tracheal basal cells characteristically express cytokeratin 5 (KRT5), while only a limited subset expresses cytokeratin 14 (KRT14). Interestingly, KRT14 is upregulated in mouse lung basal cells in response to naphtalene-injury [16]. As such, ongoing evidence highlights a role for KRT5^+^KRT14^+^ basal cells in post-injury regeneration of the mouse lung [6, 12–14].

Details about definitive basal cell subpopulations, however, remain to be elucidated, in particular in the human lung. In this context, basal cell subsets expressing distinct keratin (KRT) isoforms have been described [17] and recent evidence suggests alterations in KRT abundance and expression in lung disease with features of diffuse alveolar damage [18, 19]. Increased KRT5 and KRT14 expression has also been reported in the alveolar regions in idiopathic pulmonary fibrosis (IPF) [19]. Yet, the distinct quantitative and spatial abundance of KRT5^+^ and KRT14^+^ cells to IPF is unknown.

To this end, we sought to investigate and quantify the distribution of KRT5^+^ and KRT14^+^ cell populations in human lungs, obtained from healthy donors or IPF patients. We provide here, for the first time, a quantitative analysis of the distribution of KRT5^+^ and KRT14^+^ single- and double-positive cell populations in the healthy human lung. Importantly, we describe dramatic changes in the distribution and morphology of these cells in IPF. Finally, we seek to characterize their differentiation potential by fluorescent co-staining of these populations with well-accepted epithelial differentiation markers, such as acetylated tubulin, Mucin 5B, or Clara Cell 10 kDa Protein (CC10) in IPF.

## Methods

### Human lung material

Resected human lung tissue and explant material was obtained from the bioarchive at the Comprehensive Pneumology Center (CPC). Biopsies were obtained from 6 healthy donors and 5 IPF patients (UIP pattern, mean age: 57,6 ± 3,25, 3 males, 2 females). All participants gave written informed consent and the study was approved by the local ethics committee of Ludwig-Maximilians University of Munich, Germany (333-10). For staining, human lung tissue was fixed in 4 % PFA prior to paraffin embedding. The 4 μm-sections were prepared with a microtome (Hyrax M 55, Zeiss) and mounted on Superfrost slides.

### Isolation of primary human bronchial epithelial cells

Basal cells were isolated from bronchial tissue (>2 mm) resected from the peripheral tumor region of otherwise normal healthy lungs. For this, the tissue was longitudinally cut, washed 3 times in MEM, supplemented with L-glutamine (2 mM) and pen/strep (100 U/ml, 100 μg/ml), and digested with Pronase E (1 mg/mL) in MEM with L-glutamine and pen/strep for 20 h at 4 °C under constant agitation. The next day, the epithelium was scraped off using a scalpel, cells further separated with an 18G and 25G needle and collected by centrifugation at 300 × g for 5 min. Isolated cells were suspended in BEGM medium (Lonza; Wokingham, UK), seeded onto rat-tail collagen type I (Sigma-Aldrich; St. Louis, MO) coated dishes, and incubated at 37 °C in a humidified incubator with 95 % air and 5 % CO_2_. Cells which have reached 80 % confluence were detached using the Clonetics™ ReagentPack™ for subculturing (Lonza) and 1×10^4^ cells/cm^2^ were seeded onto collagen type I coated coverslips. Two days later, cells were washed with HBSS, fixed with 4 % PFA for 15 min and stored in HBSS at 4 °C until analysis.

### Dual immunofluorescence analysis

All tissue stainings were performed on 4 μm-sections of lung tissue. Sections were cleared of paraffin by incubation at 60 °C (1 h) and subsequent xylene washes. Sections were then rehydrated using 100 % ethanol, followed by distilled water. Antigen retrieval was performed in 0.01 M Citric Acid (pH 6.0) in a decloacking chamber for 30 s at 125 °C. After washing in distilled water, slides were blocked for 1 h at room temperature using 5 % BSA in PBS (blocking solution, BS). Primary antibodies were diluted in BS and applied to sections overnight, at 4 °C. The primary antibodies and their dilutions used to identify the cell types indicated in Table 1 were as follows: α-Cytokeratin 5 (both 1:250, clones EP1601Y or 2C2, respectively ab52635 or ab128190, Abcam), α-Cytokeratin 14 (1:200, clone LL002, ab7800, Abcam), α-p63 (both 1:25, clone 4A4, sc-8431, Santa-Cruz; or clone EPR5701, ab124762, Abcam), α-aquaporin 5 (1:100, clone C-19, sc-9891, Santa-Cruz), α-Pro-Surfactant Protein C (1:100, polyclonal, AB3786, EMD Millipore), anti-CC10 (1:100, clone H-75, sc-365992, Santa-Cruz), α-acetylated tubulin (1:250, clone AcetylK40, ab125356, Abcam), α-Mucin 5B (1:100, clone H-300, sc-20119, Santa-Cruz). Developmental pathway markers were used as follows: α-Sonic Hedgehog (1:100, clone 171018, ab50515, Abcam), α-GLI1 (1:50, polyclonal, ab49314, Abcam), α-SOX9 (1:50, polyclonal, AF3075, R&D Systems), α-HES1 (1:50, clone H-140, sc-25392, Santa-Cruz). Alexa488 (green) and Alexa586 (red)-labeled secondary antibodies (Life Technologies) were used at a 1:250 dilution in BS for 30 min. Nuclei were counterstained with DAPI and the slides mounted in fluorescent mounting medium (Dako, Hamburg, Germany). For stainings of isolated primary human bronchial epithelial cells, cells on coverslips were permeabilized with DPBS/0.1 % Triton X-100 for 5 min and blocked with BS for 1 h at room temperature. Staining for Cytokeratin 5 and 14 was performed as described above.

**Table 1:** Markers used in the study and commonly associated cell types

Marker	Cell type
Keratin 5 (KRT5)	Basal cells (airways)
Keratin 14 (KRT14)	Basal cells (regions of squamous metaplasia)
p63	Basal cells
Acetylated tubulin (acTUB)	Ciliated cells
CC10	Clara cells
Mucin 5B (MUC5B)	Goblet cells
ProSPC	Alveolar type II cells
Aquaporin 5 (AQP5)	Alveolar type I cells

### Image acquisition and analysis

All tissue images were acquired using the confocal LSM710 Microscope (Carl Zeiss; Oberkochen, Germany), coupled to the Zen2009 software, and examined by three different observers. The quantification of p63^+^, KRT5^+^ and KRT14^+^ cells was performed by counting cells in which the cytoplasm (KRT5, KRT14) or the nucleus (p63) exhibited a positive signal. The numbers of the indicated cells were normalized to the length of the corresponding region of the basement membrane, in case of conducting or distal airways, or expressed as cell/mm^2^ of analyzed tissue. Images of primary human bronchial epithelial cells were obtained using the Axiovert II (Carl Zeiss) and processed using the AxioVision 4.9 software (Carl Zeiss).

## Results

### Identification and quantification of basal cell populations in the conducting healthy airways

**Fig. 1:**
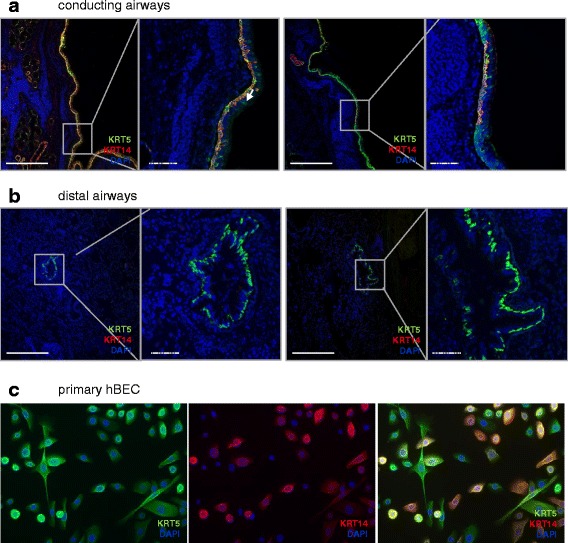
Proximal-to-distal distribution of KRT5^+^ and KRT14^+^ basal cells subpopulations in the healthy human lung. **a**, **b** Sections from healthy human lungs (6 donors) were co-stained for Keratin (KRT) 5 (*green*), KRT14 (*red*) and counterstained with DAPI (*blue*). Two representative pictures, with corresponding high magnification panels, are shown for the conducting airways (**a** *left* and *right panels*) and the distal airways (**b** *left* and *right panels*). Arrow: KRT5^+^ cell with characteristic luminal epithelial cell morphology. Full bars = 500 μm. Scattered bars = 100 μm. **c** Isolated primary human bronchial epithelial cells (hBEC) were co-stained for KRT 5 (*green*), KRT14 (*red*) and counterstained with DAPI (*blue*). The panels show: Keratin5-DAPI (*left*), Keratin14-DAPI (*middle*) and merged (*right*) images. Full bars = 500 μm. Scattered bars = 100 μm

**Fig. 2:**
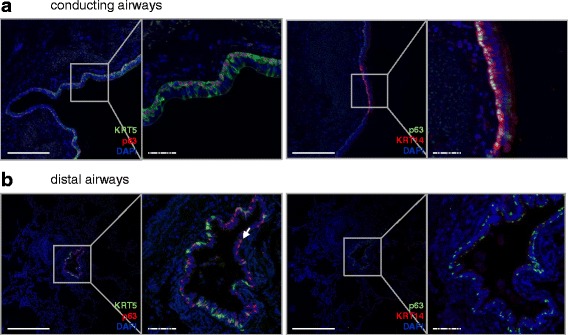
Coexpression of p63 with KRT5 and KRT14 in the healthy human lung. Sections from healthy human lungs (6 donors) were co-stained either for KRT5 (*green*) and p63 (*red*) (**a** *left*; **b** *left*), or KRT14 (*red*) and p63 (*green*) (**a** *right*; **b** *right*) and counterstained with DAPI (*blue*). One representative picture, with the corresponding high magnification panel, is shown for each staining, for the conducting (**a**) and distal (**b**) healthy airways. Arrow: KRT5^-^p63^+^ cell. Full bars = 500 μm. Scattered bars = 100 μm

**Fig. 3:**
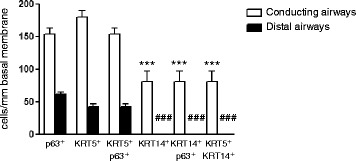
Quantification of total basal cells and KRT5^+^ and KRT14^+^ basal cell subpopulations in the healthy human lung. Cells were counted according to their positivity for p63, KRT5 or KRT14 immunostaining in conducting airways (1 donor, 2 samples, *n* = 7 assessed fields/sample) and in distal airways (5 donors, 1-3 samples/donor, *n* = 34 assessed bronchioles). Data are expressed as mean ± SEM. A 2-Way ANOVA with Bonferroni post-test revealed a significant interaction according to the type of airway (Conducting versus distal, *p* < 0.0001); all 6 quantified populations were significantly different between conducting and distal airways (***p < 0.001). Each dataset (conducting and distal) was then compared separately with a One-Way ANOVA test: ***p < 0.001 versus “Conducting KRT5^+^”, ^###^ *p* < 0.001 versus “Distal KRT5^+^”

Human healthy airways were classified into conducting and distal airways, according to the presence (conducting) or absence (distal) of cartilage and submucosal glands in the vicinity, as previously described [5, 20]. Prominent staining for KRT5 was observed all along the basement membrane of the conducting airway epithelium (Fig. 1a). All p63^+^ basal cells/mm basement membrane were KRT5^+^, while 14 % of all basal cells were KRT5^+^p63^-^ (Fig. 3). This fraction localized luminal to the basal KRT5^+^p63^+^ population in the stratified bronchial epithelium (Fig. 2a, *left panel*). A costaining for KRT5 and KRT14 revealed the existence of a KRT5^+^KRT14^+^ subpopulation that was restricted to the basal layer of the healthy conducting airways (Figs. 1a and 2a, *right panel*). This population was heterogeneously distributed, as some regions of the conducting airways were densely populated with KRT5^+^KRT14^+^ basal cells, while other regions had few KRT14^+^ cells (Fig. 1a).

All KRT14^+^ cells along the basement membrane were positive for p63 (Fig. 2a, *left panel* and Fig. 3). KRT5^+^KRT14^+^ cells represented 40 % of all KRT5+ cells, suggesting that the remaining KRT5^+^ cells were KRT5^+^KRT14^-^p63^+^ or KRT5^+^KRT14^-^p63^-^ (Fig. 3). To exclude staining bias of complex tissues, and to document these populations by an independent analysis, we isolated primary human bronchial epithelial basal cells and subjected these to immunofluorescence staining. We observed both KRT5^+^KRT14^-^ and KRT5^+^KRT14^+^ basal cell populations in primary isolated human basal cells, in similar proportions that we observed for lung tissue outlined above (Fig. 1c and *data not shown*).

### Only KRT5^+^KRT14^-^p63^+^ and KRT5^-^KRT14^-^p63^+^ basal cells populate healthy human distal airways

In comparison to the large conducting airways, the proportion of KRT5^+^p63^+^ basal cells decreased dramatically (30 cells/mm basal membrane) in the distal airways of the healthy lung (Fig. 3). Here, 40 % of p63^+^ cells did not express KRT5, demonstrating the existence of a KRT5^-^p63^+^ basal cell population. Importantly, we never observed KRT14 staining in normal distal airways, suggesting the complete absence of this population in the healthy lung (Fig. 1b).

### Distribution and nature of basal cell subpopulations in conducting airways and the distal IPF lung

**Fig. 4:**
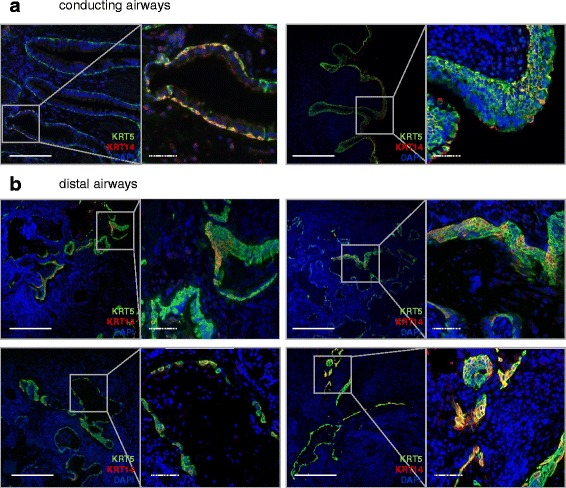
KRT5^+^ and KRT14^+^ basal cell subpopulations in the IPF lung. Sections from IPF human lungs (5 patients) were co-stained for KRT5 (green), KRT14 (red) and counterstained with DAPI (*blue*). **a** Two representative pictures, with corresponding high magnification panels, are shown for the conducting airways. **b** 4 representative pictures, with corresponding high magnification panels, illustrate the distribution patterns (metaplastic, simple and pod-like) of KRT5^+^ and KRT14^+^ basal cell subpopulations in the distal IPF lung. Full bars = 500 μm. Scattered bars = 100 μm

**Fig. 5:**
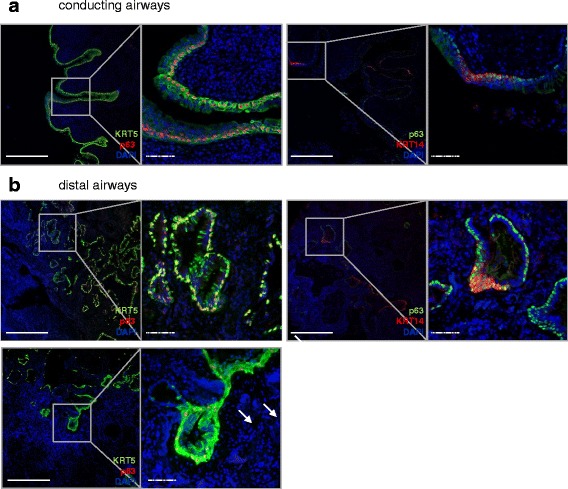
Coexpression of p63 with KRT5 and KRT14 in the IPF lung. **a**, **b** Sections from IPF human lungs (5 patients) were co-stained either for KRT5 (*green*) and p63 (*red*) (**a** *left*; **b** *left*), or KRT14 (*red*) and p63 (*green*) (**a** *right*; **b** *right*) and counterstained with DAPI (*blue*). One representative picture, with the corresponding high magnification panel, is shown for each staining, for the conducting (**a**) and distal (**b**) healthy airways. **c** Characteristic KRT5^+^ pod, stained for KRT5 (*green*) and p63 (red). Arrow: KRT5^+^p63^-^ cell. Full bars = 500 μm. Scattered bars = 100 μm

**Fig. 6:**
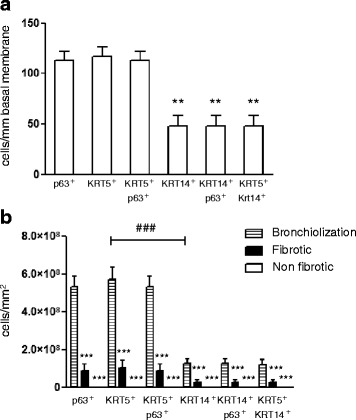
Quantification of total basal cells and KRT5^+^ and KRT14^+^ basal cell subpopulations in the IPF lung. **a**, **b** Cells were counted according to their positivity for p63, KRT5 or KRT14 immunostaining in (**a**) conducting airways (1 donor, 2 samples, *n* = 7 assessed fields/sample) and (**b**) in distal regions (5 donors, 1-3 samples/donor, *n =* 34 assessed fields in bronchiolized regions (striped bars), *n* = 24 assessed fields in fibrotic regions (black bars), *n =* 11 assessed fields in non fibrotic regions (white bars)). Data are expressed as mean ± SEM. **a** A 1-Way ANOVA revealed a significant interaction (*p* < 0.0001). The data were further analyzed for significance with a Dunn’s post-test (**p < versus “KRT5^+^”). **b** A 2-Way ANOVA with Bonferroni post-test revealed a significant interaction according to the type of remodeled region (*p* < 0.0001). Each dataset (bronchiolization, fibrotic, non fibrotic) was then compared separately with a 1-Way ANOVA test, with a Dunn’s post-test: ***p < 0.001 versus the neighboring “Bronchiolization” column, ^###^ *p* < 0.001 between “Bronchiolization KRT5^+^” and “Bronchiolization KRT14^+^”

Similar to the healthy human lung, a KRT5^+^KRT14^-^p63^+^ (112 cells/mm basement membrane) and a rare KRT5^+^KRT14^-^p63^-^ population were present in conducting airways of IPF patients (Fig. 4a). Similarly, 41 % of the total KRT5^+^p63^+^ population expressed KRT14 (Fig. 6a). In the distal IPF lung, however, the profile of KRT5 and KRT14-expressing cells was dramatically altered compared with healthy lungs (Fig. 4b). While KRT5^+^ cells were only present in the basal layers of healthy distal airways, this cell population was more widely distributed in the IPF interstitium. This KRT5^+^ progenitor population was present in fibrotic areas, mostly as pods (Fig. 6b), and dominant in bronchiolized areas (85 % of all the KRT5^+^ cells found in the distal IPF lung). KRT5^+^ cells exhibited a cuboidal or elongated morphology in IPF airways, while they were pyramidal in healthy distal airways (Fig. 4b). KRT5^+^ were frequently organized into metaplastic epithelial-like structures (Fig. 4b, *two upper panels*), single epithelial layers with distinct cell morphology (Fig. 4b, *lower left panel*), or grouped in clusters or pods (Fig. 4b, *lower right panel*). While almost all KRT5^+^ cells were p63^+^ (92 %), some also were p63^-^, mostly in inner areas of central pod regions (Fig. 5c), suggesting ongoing differentiation processes similar to the KRT5^+^ luminal cells in the conducting airways.

In striking contrast to the findings observed in healthy lungs, a KRT5^+^KRT14^+^ population emerged in the distal IPF lung, and accounted for a significant part of the total KRT5^+^ population (24 % in the bronchiolized areas, and 30 % in the fibrotic areas) (Fig. 6b). These cells were frequently detected in areas of basal cell metaplasia and mostly grouped together in clusters, suggesting clonal expansion.

### KRT5^+^ cells, but not KRT14^+^ cells, coexpress epithelial cell differentiation markers in the distal IPF lung

**Fig. 7:**
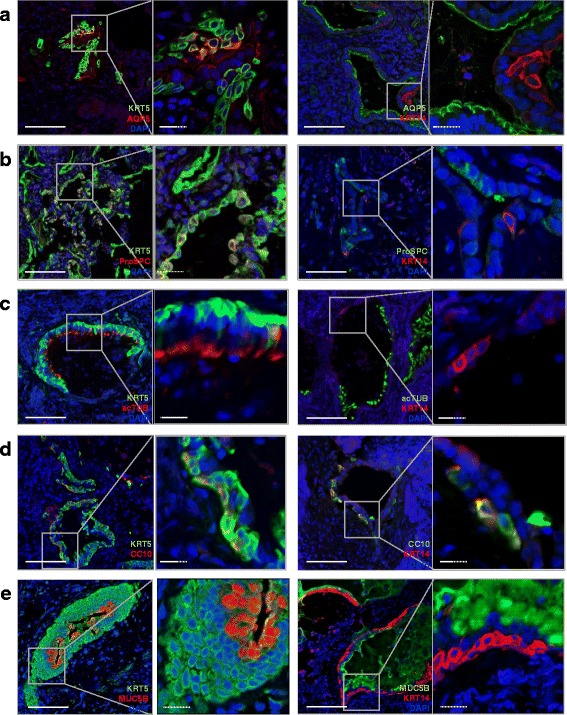
Costaining of KRT5 and KRT14 with alveolar and bronchial epithelial differentiation markers in the distal IPF lung. Human distal IPF lungs (5 patients) were costained for KRT5 or KRT14 and AQP5 (Alveolar type 1 cell marker), ProSPC (Alveolar type 2 cell marker), acTUB (ciliated cell marker), CC10 (Clara cell marker) and Mucin 5B (secretory cell marker). One picture with the corresponding high magnification is shown for each staining. **a** Left: KRT5 (*green*) and AQP5 (*red*). Right: KRT14 (*red*) and AQP5 (*green*). **b** Left: KRT5 (*green*) and ProSPC (*red*). Right: KRT14 (*red*) and ProSPC (*green*). **c** Left: KRT5 (*green*) and acTUB (*red*). Right: KRT14 (*red*) and AQP5 (*green*). **d** Left: KRT5 (*green*) and CC10 (*red*). Right: KRT14 (*red*) and CC10 (*green*). **e** Left: KRT5 (*green*) and MUC5B (*red*). Right: KRT14 (*red*) and MUC5B (*green*). Full bars = 100 μm. Scattered bars = 50 μm

In order to assess the differentiation potential of KRT5^+^KRT14^-^ and KRT5^+^KRT14^+^, costainings of KRT5^+^ or KRT14^+^ cells with a variety of alveolar or bronchiolar differentiation markers were performed (Fig. 7). Clusters of KRT5^+^ cells in the distal IPF lung exhibited co-expression of aquaporin (AQP) 5 or Pro-Surfactant Protein C (SPC), representing AT1 or AT2 markers, respectively (Fig. 7a, b). While all KRT5^+^ cells exhibited co-expression of such differentiation markers, KRT14^+^ cells, in striking contrast, did not co-express any of the known differentiation markers. In addition, KRT14^+^ cells did not even localize in proximity of AQP5^+^ or ProSPC^+^ cells. Thus, our results suggest the existence of a KRT5^+^KRT14^-^ population in the IPF distal lung, which potentially exhibits differentiation capacity into a proper alveolar epithelial cell phenotype (AT1 or AT2 cells), whereas KRT5^+^KRT14^+^ cell did not exhibit this phenotype.

We also characterized the hyperplastic bronchiolar lesions in IPF. Layers composed of KRT5^+^ basal cells in the distal IPF pods presented in close proximity of AcTub^+^-ciliated cells (Fig. 7c, *left panel*) or CC10^+^-club (Clara) cells (Fig. 7d, *upper left panel*). Interestingly, the presence of KRT14^+^ cells within the basal layer was associated with an absence of differentiated ciliated or Clara cells in the luminal layer, similar to our observation of AQP5 staining (Fig. 7c, *upper right panel* and Fig. 7d, *upper right panel*). Importantly, CC10 was found to be coexpressed in KRT5^+^ and KRT14^+^ cells, which lacked the morphology of differentiated Clara cells (Fig. 7d). In some hyperplastic epithelial structures, KRT5, but not KRT14, costained with Mucin (MUC) 5B in luminal layers (Fig. 7e, *left panel*). KRT14^+^ cells, on the other hand, were confined to the basal layer in which secreted MUC5B filled bronchiolar structure (Fig. 7e, *right panel*).

**Fig. 8:**
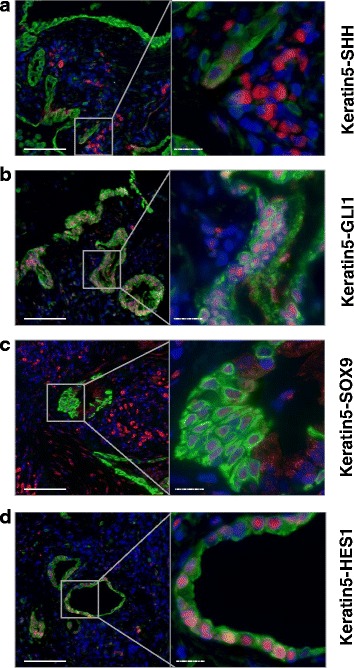
Costaining of KRT5 with developmental pathway markers in the distal IPF lung. **a**, **b**, **c**, **d** Sections from IPF human lungs (5 patients) were co-stained for KRT5 (*green*) and (**a**) SHH (*red*), (**b**) GLI1 (*red*), (**c**) SOX9 (*red*), and (**d**) HES1 (*red*), and counterstained with DAPI (*blue*). One representative picture, with the corresponding high magnification panel, is shown for each staining. Full bars = 100 μm. Scattered bars = 50 μm

To further assess whether these basal cell progenitors exhibited evidence of reactivation of developmental signaling pathways in IPF, we co-stained KRT5 + -basal cells with Sonic Hedgehog, GLI1, SOX9, or HES1 (Fig. 8). Sonic Hedgehog failed to costain in KRT5^+^ cells (Fig. 8a), while its target transcription factor (GLI1) localized to the nucleus of most of the KRT5^+^ cells (Fig. 8b). SOX9 and HES1 stained the nuclei of some KRT5^+^ cells (Fig. 8c, d), indicating the presence of KRT5^+^SOX9^+^, KRT5^+^SOX9^-^, KRT5^+^HES1^+^ and KRT5^+^HES1^-^ subpopulations amongst the IPF basal cell progenitors.

## Discussion

We show, for the first time, a proximal-to-distal quantification of bronchial basal cell subtypes in the human lung, characterized by various combinations of p63, KRT5, and KRT14 expression. While several combinations of KRT5^+/-^KRT14^+/-^p63^+/-^ basal cells populate the human airways in distinct locations, the total absence of a KRT5^+^KRT14^+^p63^+^ population in the healthy distal lung is remarkable. In IPF, the overall amount and distribution of the KRT5^+^p63^+^ cells is dramatically modified, and a frequent KRT5^+^KRT14^+^p63^+^ population emerges in the distal IPF lung. While KRT14^-^ cells co-expressed alveolar and bronchial epithelial differentiation markers, KRT14^+^ cells exhibit a metaplastic phenotype and failed to costain with any of these markers. This strongly suggests that the KRT5^+^KRT14^+^p63^+^ cell type identified in the distal IPF lung is functionally different from the KRT5^+^KRT14^-^p63^+^ cell population.

The decrease in basal cell numbers along the proximal-distal axis reported herein is consistent with the profile reported 27 years ago by Boers et al. [21]. In modification to those observations, we used the well-accepted basal cell marker p63 for cell quantification, which gives a more precise estimation of total basal cell numbers compared with morphological measures alone [4]. We sought to characterize and quantify airway epithelial progenitors in the human lung, as such data is missing up-to-date, while quantitative investigations have been performed for KRT5^+^KRT14^-^ and KRT5^+^KRT14^+^ populations in the mouse trachea [22]. Evidence of the expression of KRT5, but not KRT14, by human basal cells came from colony forming-basal cells, isolated from the proximal or distal human lung [14]. The expression of KRT14 in human basal cells was observed in basal layers of bronchospheres formed by basal cells from human lungs [6]. In addition, early studies also described KRT14 expression in basal cells in conducting healthy human airways, although without any evidence of KRT5 expression [17]. Thus, our study now provides evidence that 1) KRT5^+^KRT14^-^p63^+^ and KRT5^+^KRT14^+^p63^+^ populations coexist in similar proportions in the conducting airways, 2) KRT5^+^KRT14^+^p63^+^ cells are absent from distal human healthy lungs, 3) some KRT5^+^ cells are p63^-^, which suggests a sequential loss of basal cells markers during steady state cell renewal in the conducting airways; and 4) a rare population of KRT5^-^p63^+^ exists in the distal human lung, previously observed also by Vaughan et al. [12]. This KRT5^-^p63^+^ population of the distal airways could either express KRT17 [17] or KRT15, which is described as a marker for all basal cells in the mouse trachea [22].

IPF is a progressive, irreversible, and fatal lung disease, in which the lung epithelium is replaced by scarring tissue rich in collagens and extracellular matrix, honeycombing, and myofibroblast-rich regions. Available therapeutic approaches slow down progression of the disease, but regenerative medicine approaches would represent a promising novel option for the treatment of IPF [23]. As such, one potential option is to target basal cells, endogenous lung epithelial cells likely to have regenerative potential. While the accumulation of p63^+^ cells has been previously reported in IPF [24], distinct subpopulations were undefined. Similarly, an accumulation of KRT5^+^ cells [12], as well as KRT5^+^-KRT14^+^ cells [20] has been observed, but neither quantified nor characterized. Here, we show that, in striking contrast to normal lungs, distal IPF lungs present 1) an increased abundance of KRT5^+^ cells, 2) the emergence of a frequent KRT5^+^KRT14^+^ subpopulation, 3) morphological changes in these basal cells, and 4) the existence of characteristic patterns of KRT5^+^KRT14^-^ and KRT5^+^KRT14^+^ structures. Moreover, the quantitative analysis of these different progenitor subpopulations revealed a regional heterogeneity in the distal IPF lung. While in healthy-like non fibrotic, or in fibrotic areas, the basal cell progenitors are rarely found, they are abundant in areas of bronchiolization, suggesting the possibility of an ongoing regenerative process in these specific regions.

Current knowledge in this field is limited by the lack of an irreversible animal model more closely mimicking these features of IPF, since bleomycin-treated mice do not exhibit basal cell accumulation in the lungs [12]. To date, only the tracheal epithelium-injury model using naphtalene reliably increases the proportion of KRT14^+^ cell populations in the mouse [22]. Interestingly, upon infection with H1N1, KRT5^+^p63^+^ cells also accumulate in the distal parts of mouse lungs, forming clusters or pods [14]. In our study, we detected, amongst others, similar patterns of KRT5^+^ cells in IPF lungs. Moreover, regions of pronounced KRT5^+^ cell metaplasia in IPF samples were frequently characterized by KRT14 coexpression, suggesting hyperplastic potential of the KRT5^+^KRT14^+^ basal cell population. This is consistent with the hyperproliferative potential attributed to KRT14+ cells. Indeed, the knockdown of KRT14 in basal epithelial cells of the skin leads to cell cycle arrest [25].

In mice, reporter lines have enabled to describe the different origins of the KRT5^+^ basal cell population that arises in the mouse lung after injury. Vaughan et al. showed that the KRT5^+^ cells accumulating after H1N1 injury are mostly not of a KRT5^+^, but a lineage-negative origin. Another study showed that Clara cells dedifferentiate into p63^+^KRT5^+^ basal cells [26]. Interestingly, we observed a surprising coexpression of KRT5 or KRT14 with the Clara cell marker CC10 in the basal layers of typical bronchiolar structures in IPF. Therefore, although we cannot provide direct evidence of the origin of basal cells in IPF, a dedifferentiation of CC10^+^ cells cannot be excluded.

The dedifferentiation and transdifferentiation capacities of basal cells are strongly dependent on the type of injury [10] and their proximal-to-distal origin [14]. In the mouse, tracheal KRT5^+^ cells give rise to club (Clara) and ciliated cells under steady state or epithelial injury conditions [6]. After selective ablation of KRT5^+^KRT6^+^p63^+^ distal airway stem cells (DASC), mice failed to regenerate AT1^+^ structures after H1N1 injury [13]. The differentiation potential of human basal cells is tightly dependent on their origin and assay-specific environment [14]. In IPF, Vaughan et al. could not identify KRT5^+^SPC^+^ coexpressing cells. In contrast, in the human lung, KRT5 was frequently coexpressed with ProSPC, in particular in hyperplastic regions. Moreover, further costainings between KRT5 and alveolar/bronchial epithelial cell markers suggested a multidirectional differentiation capacity of these cells in IPF.

The fate of these basal progenitor cells in IPF is likely to be dependent on the dysregulation of numerous growth factors and cytokines, which can affect the differentiation potential of basal cells [27], but also on the adjacent ECM [28], all of which is dramatically altered in IPF. The dysregulated signaling environment changes the intracellular signaling cascades in the IPF epithelium [29], in particular with respect to aberrant activation of developmental pathways in IPF [30, 31]. During lung development, SHH, the most broadly expressed Hedgehog ligand, controls proper branching morphogenesis and patterns the lung [32]. SHH is upregulated in IPF, where it localizes to alveolar and bronchiolar epithelial cells, as previously reported by Bolaños et al. [30]. In agreement, we observed SHH staining in the distal IPF lung, but not in KRT5^+^ basal cells. Interestingly, the SHH-induced transcription factor GLI1, which is known for its involvement in stem cell renewal in non-small cell lung cancer [33], localized to the nuclei of most KRT5^+^ cells in the distal IPF lung, arguing for activation of the SHH pathway in these cells. HES1, a transcription factor activated by canonical Notch signaling, induces Clara cell differentiation in the developing lung [34]. HES1 is expressed in KRT5^+^ cells in areas of honeycombing in IPF, suggesting impaired differentiation potential into AT2 cells [12]. Our co-immunostainings for KRT5 and HES1 confirmed patchy Notch signaling activation in the KRT5^+^ subpopulation. In addition, SOX9 participates in bronchial development. Tight control of SOX9 levels is required for proper epithelial cell proliferation, since SOX9 inhibits epithelial differentiation in the developing mouse lung [35]. Upregulation of SOX9 is associated with lung cancer [36]. We observed significant numbers of KRT5^+^ cells in the IPF lung with prominent nuclear SOX9^+^ staining. Altogether, these and other findings thus suggest abnormal activation of at least three signaling pathways involved in cell proliferation and differentiation in IPF basal cells, and further indicate that the differentiation potential of the KRT5^+^ population in IPF is modified compared with quiescent healthy cells.

While KRT14^+^ cells have been reported to coexpress AT2 cell markers in DAD [18], we could not detect any costaining of KRT14^+^ with differentiation markers. The function of the KRT5^+^KRT14^+^ population therefore remains unclear. Under steady-state conditions, both KRT5^+^KRT14^-^ and KRT5^+^KRT14^+^ subpopulations are mitotic in the mouse trachea [22]. Interestingly, the increase in the number of KRT14+ cells and their contribution to the mitotic pool suggests that this subpopulation gives rise to a highly mitotic reparative pool after naphtalene injury in the mouse [22]. The hypothesis of a proliferative function of KRT14^+^ cells is reinforced by the tumorigenic potential demonstrated for KRT14^+^ cells from the skin [25], but also in the lung, where tumors spontaneously develop in knock-in mice for human KRT14 [37]. Yet, some evidence also suggests a differentiation potential for the KRT5^+^KRT14^+^ population. Indeed, after naphtalene injury, some KRT14^+^ cells had a luminal morphology in the mouse trachea, whereas the differentiating cells were mostly KRT14^-^, which indicates a quick shift in the KRT14 expression during differentiation.

Lately, new pathways determining basal cell fates after injury have been emerging in the mouse. Pardo-Saganta et al. showed that depending on the intracellular pathways activated in the basal cells, they were committed either to a ciliated or a secretory cell lineage [38]. These discoveries, combined with an accurate description of epithelial progenitor subpopulations in human healthy and diseased lung, are promising approaches for further use of lung epithelial progenitor cells for therapeutic manipulation and epithelial regeneration.

## Conclusions

This work provides a quantitative analysis of the regional distribution of KRT5^+^KRT14^+^p63^+^ and KRT5^+^KRT14^-^p63^+^ basal cell subpopulations in the healthy human lung. Dramatic changes in the amount and distribution of these cell populations are observed in IPF. In IPF, these populations express differentiated epithelial cell markers, showing that in a context of injury/disease, they are likely attempting to regenerate the epithelium. This work provides a basis for further descriptions of the molecular pathways that may control the fate of progenitor basal cells in human lung disease.

## Abbreviations

acTUB, acetylated tubulin; AQP5, aquaporin 5; AT1, alveolar Type 1; AT2, alveolar type 2; CC10, clara cell 10 kDa-protein; IPF, idiopathic pulmonary fibrosis; KRT, keratin; MUC5B, mucin 5B; ProSPC, pro-surfactant protein C
